# Triplet Loss Guided Adversarial Domain Adaptation for Bearing Fault Diagnosis

**DOI:** 10.3390/s20010320

**Published:** 2020-01-06

**Authors:** Xiaodong Wang, Feng Liu

**Affiliations:** Research Center for High-Speed Railway Network Management of Ministry of Education, School of Computer and Information Technology, Beijing Jiaotong University, Beijing 100044, China; fliu@bjtu.edu.cn

**Keywords:** unsupervised domain adaptation, Wasserstein distance, triplet loss, fault diagnosis

## Abstract

Recently, deep learning methods are becomingincreasingly popular in the field of fault diagnosis and achieve great success. However, since the rotation speeds and load conditions of rotating machines are subject to change during operations, the distribution of labeled training dataset for intelligent fault diagnosis model is different from the distribution of unlabeled testing dataset, where domain shift occurs. The performance of the fault diagnosis may significantly degrade due to this domain shift problem. Unsupervised domain adaptation has been proposed to alleviate this problem by aligning the distribution between labeled source domain and unlabeled target domain. In this paper, we propose triplet loss guided adversarial domain adaptation method (TLADA) for bearing fault diagnosis by jointly aligning the data-level and class-level distribution. Data-level alignment is achieved using Wasserstein distance-based adversarial approach, and the discrepancy of distributions in feature space is further minimized at class level by the triplet loss. Unlike other center loss-based class-level alignment approaches, which hasto compute the class centers for each class and minimize the distance of same class center from different domain, the proposed TLADA method concatenates 2 mini-batches from source and target domain into a single mini-batch and imposes triplet loss to the whole mini-batch ignoring the domains. Therefore, the overhead of updating the class center is eliminated. The effectiveness of the proposed method is validated on CWRU dataset and Paderborn dataset through extensive transfer fault diagnosis experiments.

## 1. Introduction

As one of the key components of rotating machines, the working condition of rolling bearing is critical to the safe running of the machines. Effective fault diagnosis, which aims to identify early faults and prevents system failure, could increase the safety and reliability of machinery. In the past years, a large number of intelligent fault diagnosis methods have been proposed, such as support vector machine (SVM), artificial neural network (ANN) and deep learning approaches [1]. Recently, deep learning has emerged as the most prevailing methods for fault diagnosis and health management [2].

However, most deep learning methods only work well under the assumption that enough labeled training data is available, and training and test data are drawn from the same distribution [3]. When these conditions cannot be satisfied, the performance of the deep fault diagnosis methods may significantly decline [4]. The domain discrepancy poses a major obstacle in adapting predictive models across domains. When applying the fault diagnosis model in real-world scenarios, the distribution of training and test data are often different due to the rotation speeds and load conditions of rotating machines aresubject to change during operations. Once the distribution changes, recollec the labeled training data under new distribution and retrain the model isnecessary, which is often infeasible. A commonly used approach to alleviate this domain shift problem is fine-tuning the network learned from the source domain to fit the new data distribution in the target domain [5]. Since it will be too expensive to recollect and annotate data in mechanical systems, it is impractical to fine-tuning the fault diagnosis model for the target task.

Unsupervised domain adaptation (UDA) aims at transferring of knowledge from a labeled source domain to an unlabeled target domain, and a lot of research has been carried out on this issue. Early domain adaptation approaches try to map the data into shared feature space and minimize the distance between the feature distributions of different domains. Correlation distances [6] and maximum mean discrepancy [7] are the most commonly used measurement distance. For bearing diagnosis under different working conditions, Maximum mean discrepancy (MMD) is utilized to reduce the domain discrepancy between feature representations extracted by deep neural network (DNN) [8] or sparse auto-encoder (SAE) [9]. In [10], the domain discrepancy is further reduced by multi-kernel MMD in multi layers of deep convolutional neural network. Although MMD has shown remarkable diagnosis accuracy, it brings additional computational cost since the quadratic time complexity, as shown in [11].

Recently, adversarial based adaptation approaches [12,13] have shown promising results in domain adaptation. Similar to generative adversarial networks (GAN) [14], adversarial based adaptation approaches aim to minimize the domain discrepancy through adversarial learning. They train a feature extractor and a domain discriminator to compete against each other. A domain discriminator is trained to tell which domain the sample comes from. The feature extractor is trained to confuse the domain discriminator while minimizing the classification loss. The Wasserstein distance has recently been introduced into domain adaptation of fault diagnosis and achieves competitive results [15,16]. In [15] Cheng et al. utilized Wasserstein distance to minimize distribution discrepancy through adversarial training in fault diagnosis transfer learning scenarios. Instead of minimizing the Wasserstein distance between one single layer of the neural network, In [16] Zhang et al. further proposed to minimize Wasserstein distance between multi layers of the deep neural network and achieve better accuracy on bearing fault diagnosis tasks. 

Existing work mainly concentrates on minimizing the global distribution discrepancy when learning shared feature representation across different domains. Even if the global distribution has been successfully aligned, however, the samples with the same label from different domains could still be mapped far from each other in the feature space. Some approaches have been proposed to alleviate this class-level misalignment problem [17]. One kind of approaches is to make the feature more discriminative, so as to reduce the possibility of misclassifying the samples far from the corresponding class centers [18,19]. Chen et al. [20] proposed a joint domain alignment and discriminative feature learning (JDDA) approach. The domain alignment is achieved by correlation alignment (CORAL), and center loss is imposed to samples from labelled source domain. Instead of only considering discriminative on source domain, Zhang et al. [18] proposed to impose center loss constraint to source domain and target domain, respectively. Considering no label information is available in the target dataset, the classifier trained on source domain is utilized to generate pseudo-labels for target samples. For bearing fault diagnosis, Li et al. [21] proposed a deep metric learning approach to learn a robust fault diagnosis model for domain adaptation. Better intra-class compactness and inter-class variance are achieved through representation clustering of source labeled domain.

Another approach of class-level alignment aims to further minimize the distance between the same classes from different domains which is called semantic domain alignment. Xie et al. [22] proposed to learn semantic representations by aligning labeled source centroid and pseudo-labeled target centroid. Instead of separately computing centers for source data and target data, Chen et al. [19] propose to share the class center for samples from the source domain and target domain. However, computing and updating the class center is not trivial.

**Figure 1:**
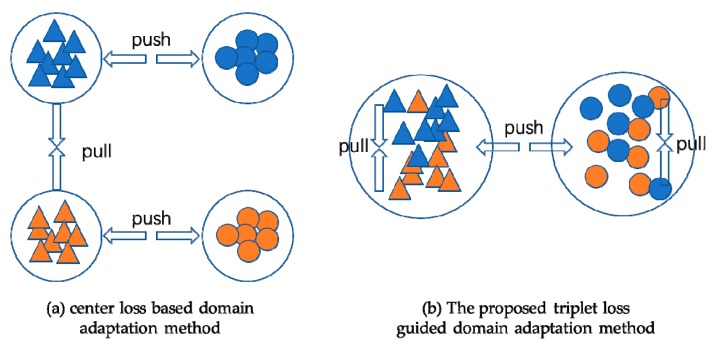
The architecture of the proposed method. Instead of computing and updating class centers for each class and reducing the distance of the same class center from different domain, the proposed TLADA method concatenates 2 mini-batches from source and target domain into a single mini-batch and imposes triplet loss to the whole mini-batch ignoring the domains.

To reduce the burden of computing and updating the class center in semantic domain alignment, in this paper, we present a two-level alignment approach for unsupervised domain adaptation of bearing fault diagnosis. Specifically, in domain-level alignment, we utilize Wasserstein distance to minimize the distribution discrepancy of both domains in the latent space. In class-level alignment, inspired by [23], we impose triplet loss to source data and target data simultaneously in each minibatch while training deep convolutional neural network. In this way, both the discriminative and domain-invariant representations could be learned, as shown in Figure 1.

The main contributions of this study are as follows:

1. We propose a novel and effective unsupervised domain adaptation approach for bearing fault diagnosis. Data-level and class-level alignment between the source domain and target domain are both considered.
2. We propose to use triplet loss to achieve better intra-class compactness and inter-class separability for samples from both domains simultaneously.
3. Extensive experiments are performed to validate the efficacy of the proposed method. In addition to transfer learning between different working conditions on CWRU dataset and Paderborn dataset, we also validate the transfer learning tasks between different sensor locations on CWRU dataset.

The remainder of this paper is organized as follows. The background of unsupervised domain adaptation, Wasserstein distance, and deep metric learning are discussed in Section 2. The proposed fault diagnosis approach is specified in Section 3. Experiments and analysis on CWRU dataset and Paderborn dataset are presented in Section 4. We close the paper with conclusions in Section 5.

## 2. Backgrounds 

In this section, unsupervised domain adaptation for fault diagnosis, Wasserstein distance and deep metric learning are introduced.

### 2.1. Unsupervised Domain Adaptation

Unsupervised domain adaptation aims to alleviate the domain shift problem by aligning the distribution between the labeled source domain and the unlabeled target domain. A common approach for domain adaptation is to map the data into a shared feature space and then employ some distance measurement to minimize the distance between the feature distributions of different domains. Maximum mean discrepancy (MMD) [24,25] measures the squared distance between the kernel embeddings of marginal distributions in the reproducing kernel Hilbert space (RKHS). Based on MMD, Pan et al. [26] proposed transfer component analysis (TCA) to minimize the discrepancy of two domains and it has been widely used in many traditional transfer learning applications. Tzeng et al. [25] introduced MMD into deep neural networks named deep domain confusion (DDC). DDC uses one adaptation layer and domain confusion loss to learn domain invariant representations. In deep adaptation network (DAN) proposed by Long et al. [27], multiple adaptation layers and multi-kernel MMD are used to further reduce the distribution discrepancy. Different than MMD, CORAL only matches the sample mean and covariance of the source and target domains, but still has high capability in domain adaptation. Then, Sun et al. [6] introduced CORAL to deep neural networks and proposed DeepCoral.

For fault diagnosis tasks, Lu et al. [8] firstly investigated MMD in deep neural networks for domain adaptation of bearing and gearbox fault diagnosis. Wen et al. [9] utilized sparse auto-encoder (SAE) to extract features and then minimize the discrepancy between features of source domain and target domain using MMD. In [10] Li et al. improved the effect of domain adaptation through multi-layer and multi-kernel MMD between domains. Except for the widely used CWRU dataset, a more practical experiment was performed on a high-speed multi-unit train bogie bearing dataset. Rather than transferring from different working conditions of the same dataset, Yang et al. [28] explored a more challenging task that transfers between different datasets, namely a laboratory bearings and a real locomotive bearings.

In contrast, adversarial learning is also widely used in domain adaptation. Many recent UDA approaches leverage deep neural networks with the adversarial training strategy, which allows the learning of feature representations to be simultaneously discriminative for the labeled source domain data and indistinguishable between source and target domains. In [12], Ganin et al. proposed a technique called domain-adversarial training of neural networks (DANN), which utilizes a gradient reversal layer and an auxiliary domain classifier to train feature extractor in an adversarial way. Tzeng et al. [13] proposed a method called adversarial discriminative domain adaption (ADDA). An encoder was trained on source samples at the first stage, then the encoder and the domain critic are trained simultaneously through minimax game until the features extracted from the source domain and target domain are indistinguishable.

In the research of adversarial based domain adaptation for bearing diagnosis, Han et al. [29] employed the DANN strategy to train fault diagnosis model for wind turbine and gearbox. In [30], Zhang et al. proposed adversarial adaptive 1-D convolutional neural networks. The architecture is in according with ADDA where two different feature extractors with partially tied weights are used. In [31], Guo et al. proposed a deep convolutional transfer learning network (DCTLN) for fault diagnosis on unlabeled data. In this method, a feature extractor and a health condition classifier are employed to learn class discriminative features, while a domain classifier and MMD based distribution discrepancy metrics are used to guide the feature extractor to learn domain invariant features.

### 2.2. Wasserstein Distance

Recently, inspired by WGAN [32], the Wasserstein distance also has been investigated in domain adaptation as a distance measurement of distribution discrepancy. The Wasserstein distance of two distributions is informally defined as the minimum cost of transforming one distribution into another. Compared with other divergences such as KullbackLeibler (KL) divergence, Wasserstein distance is continuous and differential almost everywhere, which makes it a more sensible cost function when learning distributions supported by low dimensional manifolds. Later on, Gulrajani et al. [33] proposed a new gradient penalty term to make it more robust to gradient vanishing problem. 

Shen et al. [34] proposed to utilize a discriminator to estimate empirical Wasserstein distance between the source and target samples and optimized the feature extractor network to minimize the distance in an adversarial way. In [15], Cheng et al. utilized Wasserstein distance to minimize distribution discrepancy through adversarial training in fault diagnosis transfer learning scenarios. Instead of minimizing Wasserstein distance between one single layer of the neural network, Zhang et al. [16] proposed to learn domain invariant representations through minimizing the Wasserstein distance between multi-layers of the deep neural network and achieves better accuracy on bearing fault diagnosis tasks.

### 2.3. Deep Metric Learning

Although the distributions of source and target domains could be aligned using domain adaptation methods aforementioned, the samples from different domains could still be misaligned at class level, since they mainly concentrate on minimizing the global distribution discrepancy when learning shared feature representation [22]. Even if the global distribution has been successfully aligned, the samples with the same label from different domains could still be mapped far from each other in the feature space. This class-level misalignment will have a negative effect on the generalization of domain adaptation methods.

To alleviate the class-level misalignment problem, deep metric learning is commonly used to make the feature more discriminative, so as to reduce the possibility of misclassifying the samples far from their corresponding class centers [20]. Deep metric learning aims to learn discriminative embeddings such that similar samples are nearer and different samples are further apart from each other via the deep neural network. The Euclidean distance or cosine distance could be used as the distance metric between samples. Lots of loss functions have been proposed to optimize the metric learning procedure and the most widely used loss functions are center loss [35], contrastive loss [36], and triplet loss [37].

Zhang et al. [18] introduced center loss to obtain domain-invariant and discriminative representations. The samples in the source domain and target domain could be better clustered to their corresponding centers. Considering no label information is available in the target dataset, pseudo-labels are used to calculate the class centers of the target domain. Chen et al. [19] proposed a modified center loss by which the class centers are shared between the source domain and target domain, hence the calculation for class centers could be simplified. In the target domain, the class centers are calculated using pseudo-labels and updated periodically. However, computing and updating the class center is not trivial, and the falsely pseudo labels could induce obvious bias. Instead of using pseudo-labels to match the distributions directly, Xie et al. [22] proposed to learn semantic representations by aligning labeled source centroid and pseudo-labeled target centroid. 

Triplet loss was initially introduced in face recognition tasks in order to learn a metric or an embedding space that makes the instances from the same category closer to each other than those from different categories [37]. As shown in [38], learning representations using triplet loss are superior to using pair-based loss. Inspired by this, we aim to reduce the distribution discrepancy and utilize the triplet loss to preserve the class-level relations among samples from both domains. As Equation (1), triplet loss takes triplet samples as input, which are called anchor sample, positive sample and negative sample, respectively. Triplet Loss tries to make the distance in the embedding space between the anchor sample $x_{a}$ and positive sample $x_{q}$ which belong to the same category closer than that of the anchor sample $x_{a}$ and negative sample $x_{n}$, which belong to different categories, by at least margin m.

(1) $$L_{trip}\left(\theta \right)=\sum_{\begin{array}{c} a,p,n\\ y_{a}=y_{p}\neq y_{n} \end{array}}\max\left(0,D_{a,p}-D_{a,n}+m\right)$$

## 3. Proposed Method

### 3.1. Overview

In unsupervised domain adaptation, we have labeled dataset $D_{s}={\left\{\left(x_{i}^{s},y_{i}^{s}\right)\right\}}_{i=1}^{n_{s}}$ sampled from the source domain $X^{s}$ and unlabeled dataset $D_{t}={\left\{x_{j}^{t}\right\}}_{j=1}^{n_{t}}$ sampled from the target domain $X^{t}$. The source domain and target domain share the same feature space $\left(X^{s},X^{t}\in X\right)$ but with different marginal distributions $\left(P^{s}\left(X^{s}\right)\neq P^{t}\left(X^{t}\right)\right)$. The target task is assumed to be the same as the source task. Our goal is to develop a deep neural network $f\;\;:\;\;X^{s}\to X^{t}$ that is able to predict labels for the samples from the target domain.

**Figure 2:**
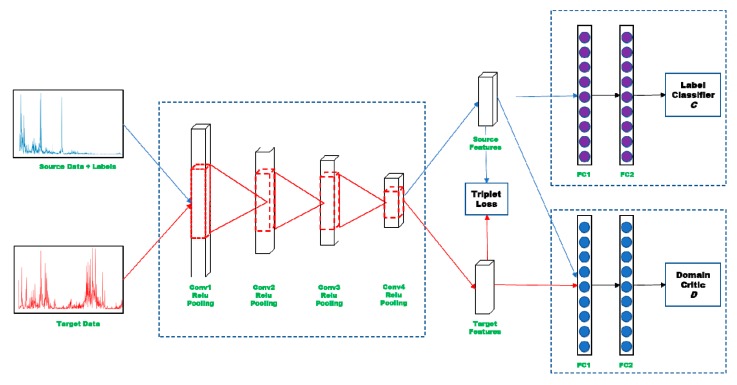
The architecture of the proposed method.

We introduce an unsupervised domain adaptation method to jointly align the distributions between the source domain and target domain in both data-level and class-level. Wasserstein distance is used to minimize the distribution discrepancy at data-level, and triplet loss is utilized to further align the distribution at class-level. The framework of the proposed method is illustrated in Figure 2. To mitigate the domain shift of different working conditions of bearing fault diagnosis by jointly aligning the two distributions, adversarial learning is performed between domain critic D and feature extractor E to minimize the Wasserstein distance, so as to align the distribution on data level, In addition, triplet loss is also imposing to source data and target data simultaneously in each minibatch while training deep convolutional neural network. Through this two-level alignment approach, both the discriminative and domain-invariant representations could be learned.

### 3.2. Domain-Level Alignment by Wasserstein Distance

To align the distribution globally using Wasserstein distance, three components are involved in this stage, namely feature extractor *E*, classifier *C* and domain critic *D*. After adversarial training between feature extractor *E* and the others, domain alignment could be achieved, and domain invariant representation could be obtained.

Given an instance $x\in R^{m}$ from either domain, the feature extractor learns a function $f_{g}:R^{m}\to R^{d}$ that maps the instance to a feature representation $h=f_{g}\left(x\right)$ with the corresponding network parameter $\theta_{g}$. A domain critic learns a function $f_{w}:R^{d}\to R$ that maps the feature representation to a real number with the parameter $\theta_{w}$. Then, the Wasserstein distance between two representation distributions $P_{h^{s}}$ and $P_{h^{t}}$, where $h^{s}=f_{g}\left(x^{s}\right)$ and $h^{t}=f_{g}\left(x^{t}\right)$, can be computed by:

(2) $$\begin{array}{cc} W_{1}\left(P_{h^{s}},P_{h^{t}}\right) & =\underset{{∥f_{w}∥}_{L}\leq 1}{\sup}E_{P_{h^{s}}}\left[f_{w}\left(h\right)\right]-E_{P_{h^{t}}}\left[f_{w}\left(h\right)\right]\\ & =\underset{{∥f_{w}∥}_{L}\leq 1}{\sup}E_{P_{x^{s}}}\left[f_{w}\left(f_{g}\left(x\right)\right)\right]-E_{P_{x^{t}}}\left[f_{w}\left(f_{g}\left(x\right)\right)\right] \end{array}$$

If the parameterized family of domain critic functions are all 1-Lipschitz, then we can approximate the empirical Wasserstein distance by maximizing the domain critic loss $L_{wd}$ with respect to parameter $\theta_{w}$:

(3) $$L_{wd}=\frac{1}{n^{s}}\sum_{x^{s}\in X^{s}}f_{w}\left(f_{g}\left(x^{s}\right)\right)-\frac{1}{n^{t}}\sum_{x^{t}\in X^{t}}f_{w}\left(f_{g}\left(x^{t}\right)\right)$$

When optimizing Equation (3) under constrain of 1-Lipschitz, a common approach is to enforce gradient penalty $L_{grad}$ for the domain critic parameter $\theta_{w}$, instead of using weight clipping method for the parameter $\theta_{w}$, which may cause gradient vanishing problem:

(4) $$L_{grad}\left(\hat{h}\right)={\left({∥\nabla_{\hat{h}}f_{w}\left(\hat{h}\right)∥}_{2}-1\right)}^{2}$$

where the feature representations $\hat{h}$ at which to penalize the gradients are defined not only at the source and target representations, but also at the random points along the straight line between source and target representation pairs. The optimization of domain critic *D* is as follows:

(5) $$\max_{\theta_{w}}\left\{L_{wd}-\rho L_{grad}\right\}$$

where *ρ* is the balancing coefficient. 

After training the domain critic *D*, we optimize the feature extractor *E* and classifier *C* during the adversarial training. The optimization goal of *E* is to minimize the Wasserstein distance with respect to parameter $\theta_{g}$ while keeping the parameters of *D* fixed:

(6) $$\min_{\theta_{g}}\max_{\theta_{w}}\left\{L_{wd}-\rho L_{grad}\right\}$$

The classifier *C* with the parameter $\theta_{c}$ will be optimized on labeled samples from the source domain. The classifier is a multi-layer fully connected network, ends with a Softmax layer with the size dependent on the classification task. The optimization function for classifier *C* is defined as:

(7) $$\min_{\theta_{C}}L_{c}=\sum_{\left(x_{i},y_{i}\right)\in \left(X_{s},Y_{s}\right)}H\left(C\left(E\left(x_{i}\right)\right),y_{i}\right)$$

where $H\left(\cdot \right)$ is the cross-entropy loss in Softmax layer, $\left(X_{s},Y_{s}\right)$ is the distribution of samples and labels in the source domain and $\theta_{c}$ are parameters of the classifier.

To sum up, the objective function in the global alignment is as Equation (8), where *λ* is the coefficient that controls the balance between discriminative and transferable feature learning and *ρ* should be set to 0 when optimizing the minimum operator.

(8) $$\min_{\theta_{g},\theta_{c}}\left\{L_{c}+\lambda \max_{\theta_{w}}\left[L_{wd}-\rho L_{grad}\right]\right\}$$

### 3.3. Class-level Alignment with Triplet Loss

Given 2 mini-batches of samples from the source domain and target domain, we compose triplet training samples using online hard negative mining strategy [37]. We first pseudo-label the mini-batch from the target domain and then concatenate two mini-batches into one. In the online triplet construction, the positive pairs are constructed using all images from the same class. For each positive pair, we randomly choose one negative sample if the negative sample is closer to the anchor point than the positive sample. The loss being minimized is then:

(9) $${ℒ}_{trip}=\sum_{i}^{N}{\left[\|f\left(x_{i}^{a}\right)-f\left(x_{i}^{p}\right){\|}_{2}^{2}-\|f\left(x_{i}^{a}\right)-f\left(x_{i}^{a}\right){\|}_{2}^{2}+m\right]}_{+}$$

The objective function in the class-level alignment is as follow

(10) $$\underset{\theta_{g},\theta_{c}}{\min}\left\{{ℒ}_{c}+\lambda_{1}\underset{\theta_{w}}{\max}\left\{{ℒ}_{wd}\right\}+\lambda_{2}{ℒ}_{tri}\right\}$$

where *λ*_1_ and *λ*_2_ are balancing coefficients.

The algorithm details of the proposed method are described in Table 1.

## 4. Experiments

**Figure 3:**
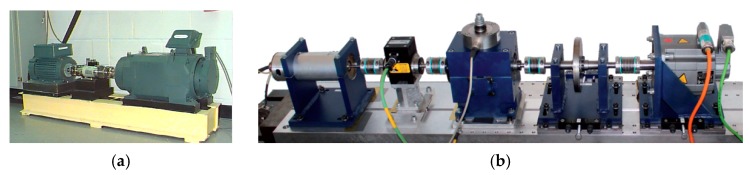
Test rig used in this paper. (**a**) CWRU testbed; (**b**) Paderborn testbed.

We evaluate our method on CWRU rolling bearings dataset and Paderborn dataset under different loads. Additional, unsupervised domain adaptation between different sensor locations is performed on CWRU dataset. The testbeds of CWRU and Paderborn are shown in Figure 3.

### 4.1. Implementation Details

The detail of the network architecture is shown in Table 2. It consists of four 1-D convolutional layers, following the rectified linear units (ReLU) activation function, and a dropout layer. The representation is then flattened and passed to classifier, domain critic, and triplet to calculate the classification loss, Wasserstein distance, and triplet loss, respectively.

To validate the performance of the proposed method, we compare our method with the Wasserstein distance-based adversarial domain adaptation approach (WDGRL) and deep learning-based domain adaptation methods. To be fair, the neural network used in our method and the compared deep learning methods are kept the same.

- Wasserstein distance guided representation learning (WDGRL) proposed by Shen et al. [34]. Wasserstein distance of representations learned from feature extractor is minimized to learn domain-invariant representations through adversarial learning.
- Deep convolutional transfer learning network (DCTLN) proposed by Lei et al. [31]. Both adversarial learning and MMD loss are employed to minimize the domain discrepancy.
- Transfer component analysis (TCA) proposed by Pan et al. [26].
- CNN: The neural network trained on labeled data from the source domain is used to classify the target domain directly without domain adaptation.
- DeepCoral proposed by Sun et al. [6]. Mean and covariance of feature representations are matched to minimize domain shift.
- Deep domain confusion (DDC) proposed by Tzeng et al. [25]. DDC uses one adaptation layer and domain confusion loss to learn domain invariant representations.
- Deep adaptation network (DAN) proposed by Long et al. [27]. Feature distributions are aligned through minimizing multi-kernel MMD distance between domains.

The reported experimental results are averaged by 10 trials to reduce the effect of randomness, and the mean values are provided. All the experiments are implemented using Pytorch and were running on NVIDIA GTX 2060 GPU. The source code is available at https://github.com/schwxd/TLADA.

### 4.2. Case 1: Results and Analysis of CWRU Dataset

In case 1, we use the public fault bearing dataset provided by Case Western Reserve University (CWRU) Bearing Data Center to evaluate the proposed method. In this study, the vibration signals recorded at 12,000 samples/second (Hz) for the drive-end bearings and fan-end bearings are used. For experiments between different working conditions of drive end, healthy condition and three fault categories (ball fault, inner raceway fault, and outer raceway fault) with three different fault depth (0.007, 0.014, 0.021 inches) are used, which make the experiments 10-category classification tasks. For experiments between different sensor locations, the samples are collected from healthy condition and three fault categories with two different fault depth (0.007, 0.021 inches), but we ignore the variance of working conditions and fault depth in this task, which make the experiments 4-category classification tasks (Healthy, Inner Race, Outer Race, Ball).

#### 4.2.1. Dataset and Implementation

We mainly fellow the experimental setup in [39] where each class has 1000 samples. The samples are generated from sensory vibrational data using an adjustable sliding window frame method to augment the dataset. The length of each sample is 2048, and the fast Fourier transform (FFT) is applied to each sample to obtain the frequency spectrum. Since the frequency spectrum is symmetric, only the first half of the spectrum is kept. Hence, the feature dimension of each sample is 1024. 

**Figure 4:**
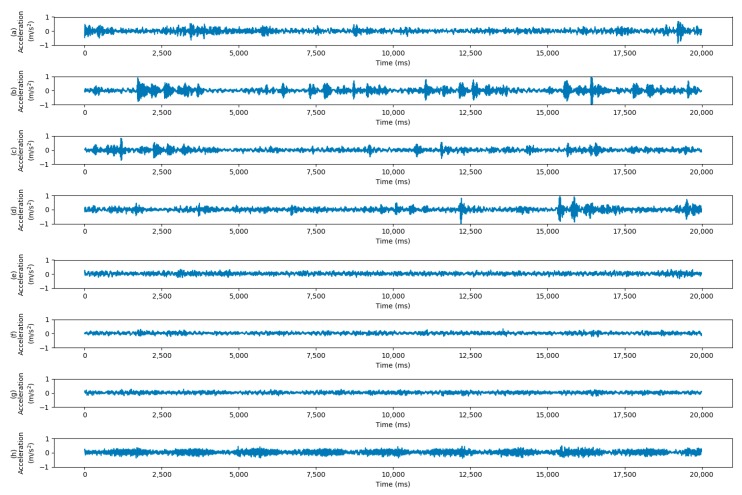
Collected signals of Ball Fault with fault depth 0.014 inch under different working conditions. The horizontal axis represents the time and the horizontal axis are the acceleration data. Four vibration signals under load conditions of 0, 1, 2, 3 from the drive end are shown in (**a**–**d**), respectively. Four vibration signals under load conditions of 0, 1, 2, 3 from the fan end are shown in (**e**–**h**), respectively.

The dataset consists of four working conditions with different motor load and rotating speed, i.e., Load0 = 0 hp/1797 rpm, Load1 = 1 hp/1772 rpm, Load2 = 2 hp/1750 rpm and Load3 = 3 hp/1730 rpm. When transferring from different load conditions, all 4 load conditions are used to perform 12 transfer scenarios. When transferring from different sensor locations, 2 transfer scenarios (DE -> FE and FE -> DE) are performed. When transferring from dataset A to dataset B, all samples of A and half samples of B are used for training, and the models are tested on another half samples of B. The description of CWRU dataset in use is shown in Table 3. The vibration signals of different working conditions are shown in Figure 4. 

The hyperparameters used in the experiment are as follows. Learning rates of classifier and domain critic are *α*_1_ = 1 × 10^−4^ and *α*_2_ = 1 × 10^−4^. The gradient penalty *ρ* is 10. Coefficient *λ*_1_ and *λ*_2_ are 1.0 and 0.2, respectively. For MMD (Coral) based methods, the coefficient between classification loss and MMD (Coral) loss is chosen from {0.01, 0.1, 1.0, 10, 100}. For Wasserstein based methods (WDGRL and TLADA), the coefficient *λ*_1_ is chosen from {0.1, 0.2, 0.5, 0.8, 1.0, 2.0}.

#### 4.2.2. Results and Analysis

Table 4 shows the results of domain adaptation tasks on CWRU dataset. For transfer tasks between different working conditions, TLADA achieves 100% accuracy on 3 tasks and has an average 98.48% accuracy overall. For tasks having a larger margin between working conditions like ‘DE0 -> DE3’, the compared methods declined, while the Wasserstein based methods still have high accuracy. For the more complicated transfer tasks between different sensor locations, the results show a significant decline compared with results between different working conditions. Specifically, the result of WDGRL transferring from drive end to fan end drops to 61.02% while our method still has accuracy of 64.08%. 

**Figure 5:**
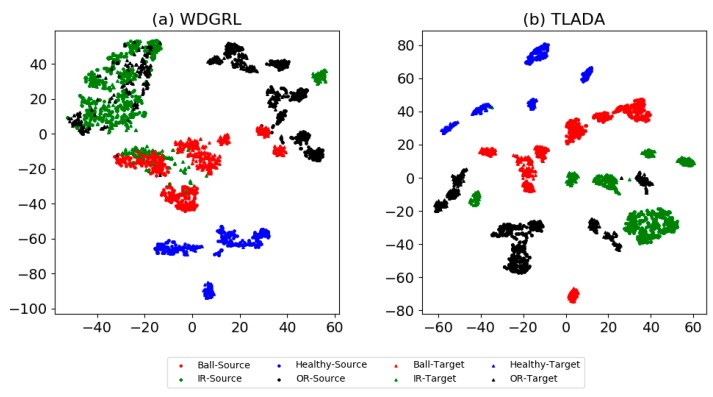
t-SNE results of task ‘FE -> DE’ on CWRU dataset through method (**a**) WDGRL, (**b**) TLADA. To better inspect the class-level alignment between domains, we draw the features of both the source domain and the target domain into single images. Two shapes represent two domains (square for source domain and triangle for target domain), and four colors with numbers represent four classes.

**Figure 6:**
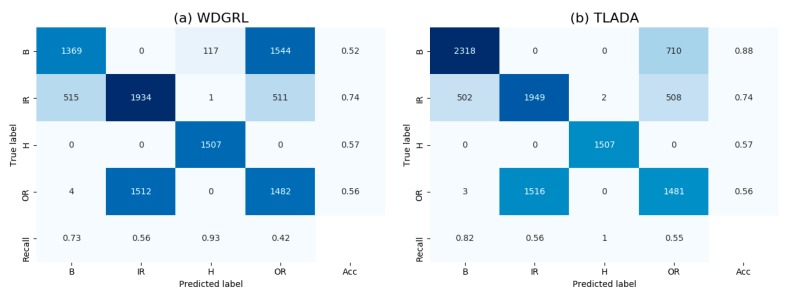
Confusion matrix results of task ‘FE -> DE’ on CWRU dataset through method (**a**)WDGRL, (**b**) TLADA. The accuracy and recall of each class are added to the matrix as well.

To better understand the effect of class-level alignment in domain adaptation, we compare the domain-invariant representations between WDGRL and TLADA via t-SNE in Figure 5, and the confusion matrix results in Figure 6. We choose the task ‘FE -> DE’ for these comparisons. As shown in t-SNE result, the class of healthy condition is clearly separated, and both accuracy and recall are 100% in the confusion matrix. The accuracy of TLADA on ‘Inner Race’, ‘Outer Race’ and ‘Ball’ is higher than WDGRL. This is consistent with the t-SNE results, where the learned feature representations of TLADA on those classes are better separated than WDGRL results. By imposing triplet loss on samples, the samples far away from their class centers, which are prone to be misclassified, are further reduced compared to WDGRL. 

### 4.3. Case 2: Results and Analysis of Paderborn Dataset

In case 2, we evaluate our method on Paderborn University bearing dataset [40]. The dataset consists of 6 healthy bearing sets and 26 damaged bearing sets. Current signals and vibration signals are sampled from the test rig. In this study, we only adopt the vibration signals which are sampled with 64kHz resolution. In addition to 12 artificially damaged bearing sets, 14 bearing sets are real damaged using accelerated life tests, which are prone to have multiple damages.

**Figure 7:**
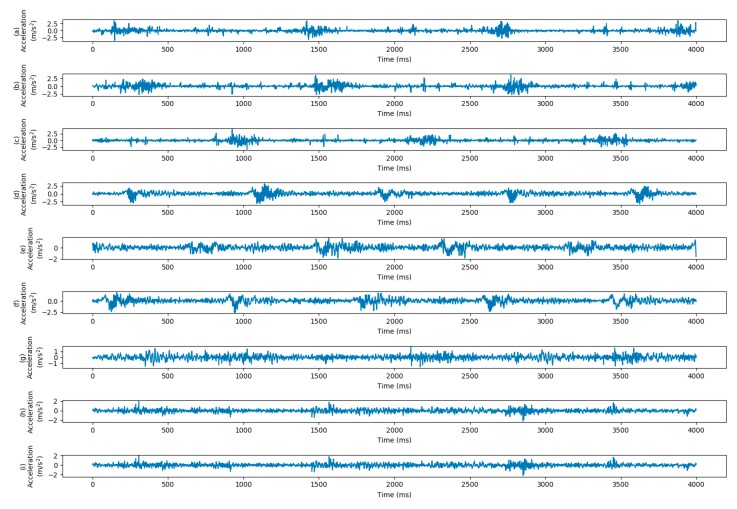
Vibration signals of Paderborn dataset under different working conditions. The horizontal axis represents the time and the horizontal axis are the acceleration data. Health signals (K001) under working conditions of 1, 2, 3 are shown in (**a**–**c**), respectively. Outer Race signals (KA04) under working conditions of 1, 2, and 3 are shown in (**d**–**f**), respectively. Inner Race signals (KI04) under working conditions of 1, 2, and 3 are shown in (**g**–**i**), respectively.

In this study, we adopt 5 healthy bearing sets and 10 real damaged bearing sets to evaluate our method. Since 5 of damaged bearings are in the inner race and 5 are in the outer race, the experiment is a 3-way classification task. The healthy bearing sets mainly differ in the operating hours, as shown in Table 5. The real damaged bearing sets differ in many ways, such as damage mode, damage combination, damage extent, etc. The differences between bearing sets make it a more complex task to correctly fault classification. The parameters of faulty bearing sets are detailed in Table 6. The vibration signals of different working conditions are shown in Figure 7.

#### 4.3.1. Dataset and Experiment

To validate our method in the setting of unsupervised domain adaptation, we compose 6 transfer learning tasks between 3 working conditions. The vibration data is also preprocessed under the sliding window mechanism as case 1. Since the sample rate is 64 kHz and the rotational speed is 1500 rpm, the frame length of each sample is set to 5120 to cover 2 cycles of bearing running. Each class has 4000 training samples and 1000 test samples. Fast fourier transform (FFT) is applied to each sample to obtain the frequency spectrum and the first half of the spectrum is kept. Hence, the feature dimension of each sample is 2560. The description of Paderborn dataset in use is shown in Table 7.

The hyperparameters used in case 2 experiment are as follow: Learning rates of classifier and domain critic are α_1_ = 10^−3^ and α_2_ = 10^−3^. The gradient penalty *ρ* is 10. Coefficient *λ*_1_ and *λ*_2_ is 1.0 and 0.1, respectively.

#### 4.3.2. Results

Table 8 shows the results of our experiments on the Paderborn dataset. Although diagnosis on the artificial damaged dataset is considered to be a more challenging task, the accuracy on Paderborn dataset remains high level, which may be contributed to the huge number of training data compare to CWRU dataset. Specially, all models achieved high accuracy in tasks ‘PA -> PC’ and ‘PC -> PA’, in which the load conditions have the same radial force but different load torque. In other tasks, the Wasserstein based methods (WDGRL and TLADA) have an obvious improvement over Coral-based and MMD-based methods (DAN and DDC). Specifically, for tasks ‘PB -> PA’ and ‘PB -> PC’, CNN method without domain adaptation achieved accuracy below 90%, while other domain adaptation methods have demonstrated significant improvement. For tasks ‘PA -> PB’ and ‘PC -> PB’, TLADA exceeds WDGRL by approximately 5%. Overall, among all methods, TLADA achieved the highest average accuracy, which proves the strong domain adaptation.

**Figure 8:**
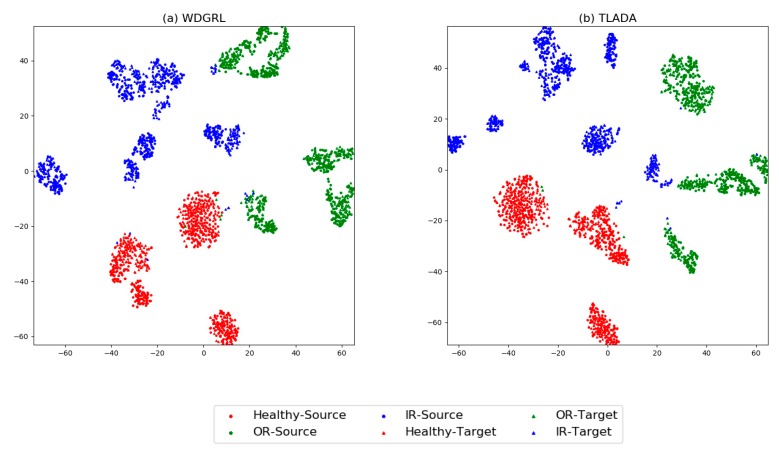
t-SNE results of task ‘PA -> PB’ through the method: (**a**) WDTRL, (**b**) TLADA. Two shapes represent two domains (square for source domain and triangle for target domain) and three colors represent three classes.

**Figure 9:**
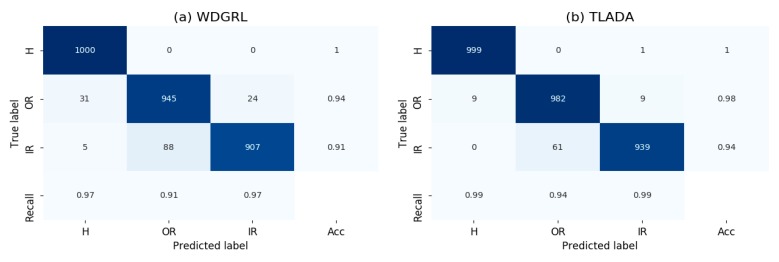
Confusion matrix of task ‘PA -> PB’ through the method: (**a**) WDGRL, (**b**) TLADA.

The visualization results produced by t-SNE method are shown in Figure 8, and the confusion matrix in Figure 9. Task ‘PA -> PB’ is chosen for comparison. Generally, the healthy condition of Paderborn dataset is not perfectly separated as CWRU dataset, and ‘Outer Race’ samples are prone to be misclassified as healthy condition. ‘Inner Race’ and ‘Outer Race’ are prone to be misclassified to each other, and this is slightly improved by TLADA. As shown in t-SNE result, more discriminative features could be achieved by TLADA compared with features learned by WDGRL.

### 4.4. Analysis

#### 4.4.1. Ablation Analysis 

**Figure 10:**
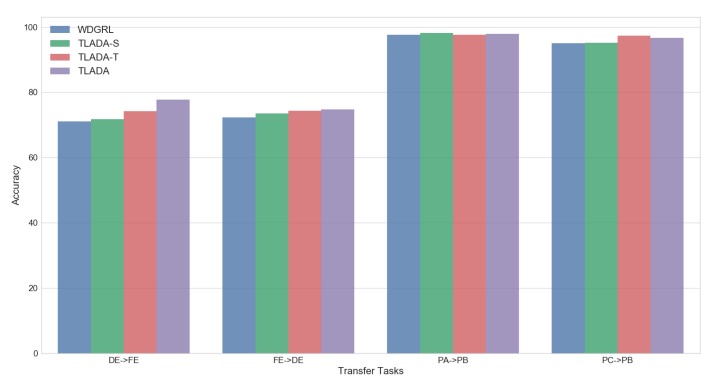
Comparison of accuracy of TLADA variants on four tasks.

To further inspect the effects of triplet loss in unsupervised domain alignment, other two variants of TLADA are built for comparison: one imposes triplet loss only on data samples from source domain called TLADA-S, and another one imposes triplet loss only on data samples from target domain called TLADA-T. We perform the experiments on four tasks: ‘DE -> FE’ and ‘FE -> DE’ on CWRU dataset, ‘PB -> PC’ and ‘PC -> PB’ on Paderborn dataset. The results are shown in Figure 10. Compared with WDGRL with no triplet loss, the other three methods have demonstrated higher accuracies, and TLADA has the most improvement among them. The TLADA-T has a better effect than TLADA-S, possibly because the classifier has been well trained on labeled samples from the source domain already. 

#### 4.4.2. Parameter Sensitive Analysis

**Figure 11:**
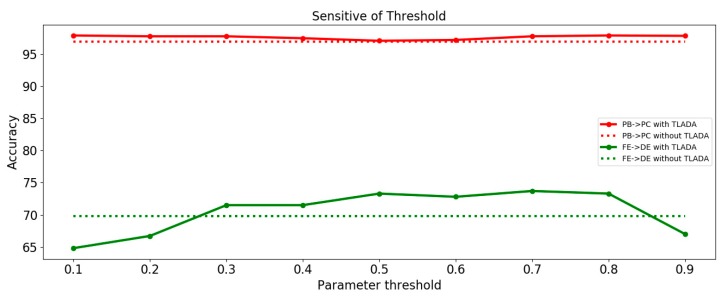
Analysis of parameter sensitivity of threshold in TLADA. Dashed lines show baseline results without TLADA.

We investigate the parameter sensitivity of threshold when assigning pseudo-labels. Figure 11 gives an illustration of the variation of transfer classification performance as threshold ∈ {0.1, 0.2, 0.3, 0.4, 0.5, 0.6, 0.7, 0.8, 0.9}. The comparison was performed on tasks ‘DE -> FE’ and ‘PB -> PC’. 

We can observe that the for task ‘PB -> PC’, TLADA accuracy is not much affected by variants of threshold since the majority of samples have been correctly classified. For task ‘FE-DE’, the accuracy of TLADA first increases and then decreases as the threshold varies. When the threshold is too low, more samples with falsely pseudo-labels will be involved in triplet loss, thus have a negative impact on the accuracy. When the threshold is too high, fewer samples are involved in triplet loss and class-level alignment will not work. It is suggested to use a moderate threshold value for hard transfer tasks.

#### 4.4.3. Computational Cost

The triplet loss comes with run-time complexity *O*(*N*^3^/*C*) per epoch, where *N* is the number of samples and *C* is the number of classes. In our method, we use two approaches to reduce the complexity of computation. One approach involves selecting samples using online hard triplets within each mini-batch. For example, in the experiment of Paderborn dataset, we observed that about 1000~2000 triplets are selected in each minibatch with batch size of 256, thus only a small portion of samples are selected for training. Second approach is imposing triplet loss only when the training of Wasserstein distance and the classifier have been stabilized, since the selection of triplet samples of target domain depends on the pseudo-labels. At the beginning of model training, the model is not discriminative enough and the predicted labels have low confident. In the experiment we found that the triplet loss could be enabled during the last 20 epochs of training, which not only aligned the distribution at class-level, but also not bring heavy burden of computation. The time cost of each method are listed in Table 9. All the methods run 200 epochs, and triplet loss of TLADA is imposed on last 20 epochs. From the results, the time cost of TLADA takes about twice of DCTLN and WDGRL, but less than the time cost of DAN method.

## 5. Conclusions

In this paper, we propose a triplet loss guided adversarial domain adaptation method for bearing fault diagnosis. We match distribution at domain-level using Wasserstein distance, as well as class-level using pseudo-labeling and triplet loss. We use triplet loss to guide the feature extractor to preserve class information for target samples in aligning domains. Experiments on two different bearing diagnosis scenarios verify the efficacy of our proposed approach.

In the future, we plan to eliminate the effect of falsely pseudo-labels in the target domain.

## Figures and Tables

**Table 1:** Algorithm details of the proposed method.

Algorithm: TLADA
Require: source data $X^{s}$; target data $X^{t}$; minibatch size *m*; critic training step *n*; learning rate for domain critic *a*_1_; learning rate for classification and feature learning *a*_2_;
Initialize feature extractor, domain critic, classifier with random weights $\theta_{g},\;\;\theta_{w},\;\;\theta_{c}$ repeat Sample minibatch ${\left\{x_{i}^{s},y_{i}^{t}\right\}}_{i=1}^{m},\;\;{\left\{x_{i}^{t}\right\}}_{i=1}^{m}$ from $X^{s}$ and $X^{t}$ for t=1,…,n do $h^{s}\leftarrow f_{g}\left(x^{s}\right),\;\;h^{t}\leftarrow f_{g}\left(x^{t}\right)$ sample *h* as the random points along straight lines between $h^{s}$ and $h^{t}$ $\hat{h}\leftarrow \left\{h^{s},h^{t},h\right\}$ $\theta_{w}\leftarrow \theta_{w}+\alpha_{1}\nabla_{\theta_{w}}\left[L_{wd}\left(x^{s},x^{t}\right)-\rho L_{grad}\left(\hat{h}\right)\right]$ end for $\theta_{c}\leftarrow \theta_{c}-\alpha_{2}\nabla_{\theta_{c}}L_{c}\left(x^{s},y^{s}\right)$ $\theta_{g}\leftarrow \theta_{g}-\alpha_{2}\nabla_{\theta_{g}}\left[L\left(x^{s},y^{s}\right)+\lambda_{1}L_{wd}\left(x^{s},x^{t}\right)+\lambda_{2}L_{tri}\left(x^{s},x^{t}\right)\right]$ until $\theta_{g},\theta_{w},\theta_{c}$ converge

**Table 2:** Details of the networks used in this experiment.

Component	Layer Type	Kernel	Stride	Channel	Activation
Feature Extractor	Convolution 1	32 × 1	2 × 1	8	Relu
	Pooling 1	2 × 1	2 × 1	8	
	Convolution 2	16 × 1	2 × 1	16	Relu
	Pooling 2	2 × 1	2 × 1	16	
	Convolution 3	8 × 1	2 × 1	32	Relu
	Pooling 3	2 × 1	2 × 1	32	
	Convolution 4	3 × 1	2 × 1	32	Relu
	Pooling 4	2 × 1	2 × 1	32	
Classifier	Fully-connected 1	500		1	Relu
	Fully-connected 2	*C* ^1^		1	Relu
Critic	Fully-connected 1	500		1	Relu
	Fully-connected 2	1		1	Relu

^1^ Depending on the categories of classification tasks, *C* is 10 or 4 for CWRU dataset and 3 for Paderborn dataset.

**Table 3:** Description of CWRU dataset.

Datasets	Working Conditions	# of Categories	Samples in Each Category	Category Details
DE0	0	10	1000	Health, Inner 0.007, Inner 0.014, Inner 0.021, Outer 0.007, Outer 0.014, Outer 0.021, Ball 0.007, Ball 0.014, Ball 0.021
DE1	1	10	1000	
DE2	2	10	1000	
DE3	3	10	1000	
DE	1/2/3	4	6000	Health, Inner (0.007, 0.021), Outer (0.007, 0.021), Ball (0.007, 0.021)
FE	1/2/3	4	6000	

**Table 4:** Test results of domain adaptation tasks on CWRU dataset. (Accuracy %).

Task	TCA	CNN	Deep CORAL	DDC	DAN	DCTLN	WDGRL	TLADA
DE0 -> DE1	62.50	95.07	98.11	98.24	99.38	99.99	99.71	99.68
DE0 -> DE2	65.54	79.28	83.35	80.25	90.04	99.99	98.96	99.81
DE0 -> DE3	74.49	63.49	75.58	74.17	91.48	93.38	99.22	99.61
DE1 -> DE0	63.63	79.99	90.04	88.96	99.88	99.99	99.67	99.82
DE1 -> DE2	64.37	89.33	99.25	91.17	99.99	100	99.88	100
DE1 -> DE3	79.88	58.48	87.81	83.70	99.47	100	99.16	99.51
DE2 -> DE0	59.05	90.96	86.18	67.90	94.11	95.05	95.25	98.32
DE2 -> DE1	63.39	88.81	89.31	90.64	95.26	99.99	93.16	96.61
DE2 -> DE3	65.57	87.15	98.07	88.28	100	100	99.99	100
DE3 -> DE0	72.92	68.09	76.49	74.60	91.21	89.26	90.75	94.37
DE3 -> DE1	68.93	75.11	79.61	74.77	89.95	86.17	95.75	95.97
DE3 -> DE2	63.97	89.84	90.66	96.70	100	99.98	99.46	100
average	67.02	80.47	87.87	84.12	95.90	96.16	97.58	98.48
DE -> FE	34.37	28.42	54.14	51.38	58.67	58.74	61.02	64.08
FE -> DE	36.40	56.65	64.93	57.67	69.14	60.40	66.23	69.35
average	35.39	42.54	59.54	54.53	63.91	59.57	63.63	66.72

**Table 5:** Operating parameters of healthy bearing of Paderborn dataset [41].

Bearing Code	Bearing Name	Damage	Class	Run-in Period [h]	Radial Load [N]	Speed [min]
K001	H1	no damage	H	>50	1000–3000	1500–2000
K002	H2	no damage	H	19	3000	2900
K003	H3	no damage	H	1	3000	3000
K004	H4	no damage	H	5	3000	3000
K005	H5	no damage	H	10	3000	3000

**Table 6:** Operating parameters of damaged bearing of Paderborn dataset [41].

Bearing Code	Bearing Name	Damage	Class	Combination	Arrangement	Damage Extent	Characteristic of Damage
KA04	OR1	fatigue: pitting	OR	S	no repetition	1	single point
KA15	OR2	plastic deform: indentations	OR	S	no repetition	1	single point
KA16	OR3	fatigue: pitting	OR	R	random	2	single point
KA22	OR4	fatigue: pitting	OR	S	no repetition	1	single point
KA30	OR5	plastic deform: indentations	OR	R	random	1	distributed
KI04	IR1	fatigue: pitting	IR	M	no repetition	1	single point
KI14	IR2	fatigue: pitting	IR	M	no repetition	1	single point
KI16	IR3	fatigue: pitting	IR	S	no repetition	3	single point
KI18	IR4	fatigue: pitting	IR	S	no repetition	2	single point
KI21	IR5	fatigue: pitting	IR	S	no repetition	1	single point

IR: Inner Race Defect; OR: Outer Race Defect; S: Single Damage; R: Repetitive Damage; M: Multiple Damage.

**Table 7:** Description of the Paderborn dataset.

Dataset		Faulty Conditions			Working Conditions		
		Normal	Inner Race	Outer Race	Rotational Speed [rpm)	Load Torque [Nm]	Radial Force [N]
PA	Train	4000	4000	4000	1500	0.1	1000
	Test	1000	1000	1000			
PB	Train	4000	4000	4000	1500	0.7	400
	Test	1000	1000	1000			
PC	Train	4000	4000	4000	1500	0.7	1000
	Test	1000	1000	1000			

**Table 8:** Test accuracy of the Paderborn dataset (Accuracy %).

3-Category Task	TCA	CNN	DeepCoral	DAN	DDC	DCTLN	WDGRL	TLADA
PA -> PB	87.27	90.93	92.23	91.70	90.83	96.17	96.33	99.00
PA -> PC	99.87	99.73	99.54	99.33	99.97	99.84	99.97	100
PB -> PA	92.99	88.73	92.20	93.03	98.13	99.87	98.80	99.17
PB -> PC	92.53	84.20	95.10	91.03	97.23	99.75	97.90	99.97
PC -> PA	99.80	99.36	99.60	97.37	99.83	99.91	99.80	99.93
PC -> PB	89.71	92.80	92.93	95.00	93.37	91.32	93.23	98.67
average	93.70	92.63	95.27	94.58	96.56	97.81	97.67	99.46

**Table 9:** Comparison of computational cost. time of 200 epochs on ‘DE -> FE’ of CWRU dataset is listed (in seconds). triplet loss of TLADA is imposed on last 20 epochs.

Task	CNN	DeepCoral	DDC	DAN	DCTLN	WDGRL	TLADA
Time (seconds)	245	530	493	2543	930	823	2079
